# Monthly Summary

**Published:** 1891-08

**Authors:** 


### Monthly Summary.

HOW TO GET A TIN CAST.—Get a good plaster model, dry it, dip in melted stearine, and take two or three impressions in molding sand. Pour one of them with tin, wait a few moments, and pour the tin in the sand back into the ladle. A thin coating of tin which has cooled gives a perfect hollow cast. If too much time is allowed the hollow cast will be too thick. A little experience will show how long to wait. With a very thin saw make several cuts from the edge to the center of the ridge. Fill with plaster, and you have a plaster cast coated with tin, and the slits made allow the sections to be bent inward, and the plate will come off. If this last precaution is not taken, you are liable to fracture the plate or check the gum sections in taking off the case. With small partial cases, that come off and on the model easily in the wax, I invest without the model, and finish and fit the case to the model after vulcanizing. Then it goes into its place in the mouth without any filling.

Let me ask some young operator, who swears by a plaster model, to try my way with the next partial plate he has to make. Of, course, I do not refer to partial suction cases, as I never make any. Get a perfect impression in modeling compound, making a special tray, if necessary; dry and dip the model; make a wax plate with a strong wire or two imbedded in it; grind in the teeth, at the chair preferably; and see that the wax plate fits the model nicely in every part. Take it off and on two or three times till you feel sure you do not bend it in so doing. Invest without the model, and when it is finished and polished it will fit the model exactly and the mouth as well. If your case ever breaks, or you want to add a tooth, your model is ready. If your patient goes abroad, and breaks the case, you can mend it, and be sure it will fit, if sent by mail· and if some poor old grinder gets loose, and drops out, you can cut the corresponding tooth off the model and add one. It is sure to be right.—*Chas. Rathbun, Items.*

---

ARISTOL (called also di-thymol) results from the combination of iodine and thymol. It has been used with great success in general surgery as an antiseptic. It is an amorphous powder of a reddish-brown color, not poisonous nor caustic and has no odor. It is very soluble in ether, chloroform, benzine and the fixed oils. It is recommended for dressing root canals and capping pulps.—*Southern Dental Journal.*

